# P-718. Getting to the Heart of Respiratory Syncytial Virus Disease Burden

**DOI:** 10.1093/ofid/ofae631.914

**Published:** 2025-01-29

**Authors:** Paulina Sudnik, Edward E Walsh, Angela R Branche, Michael E Vornovitsky, Ann R Falsey

**Affiliations:** University of Rochester, Rochester, New York; University of Rochester, Rochester, New York; University of Rochester, Rochester, New York; University of Rochester, Rochester, New York; University of Rochester School of Medicine, Rochester, New York

## Abstract

**Background:**

There is increasing evidence that viral respiratory infections are associated with acute cardiovascular events. The majority of studies to date involve SARS-CoV-2 and influenza, whereas data for RSV are limited.

**Figure:**
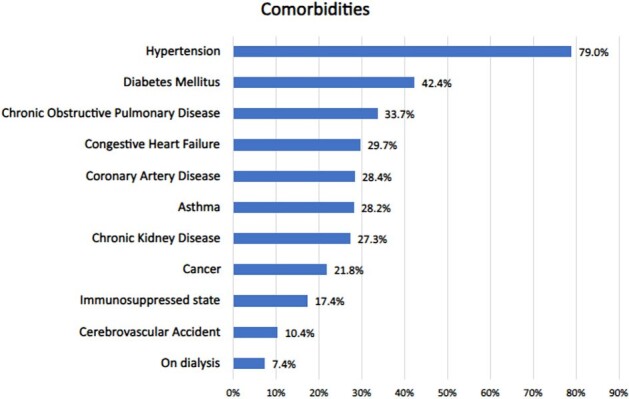

**Methods:**

We performed an analysis of electronic records from patients with documented RSV hospitalization from a prospective incidence study conducted in Rochester, NY from 2017-2020. Clinical, demographic, medical history, and cardiac risk factor data were collected. Outpatient and hospital encounters were reviewed for cardiovascular events (CVE) 6 months before RSV infection and during “high-risk” and “late-risk” periods, 28 days and 1-5 months, respectively, after RSV for a total of one year of observation. Events of interest were myocardial infarction (MI), new or exacerbation of congestive heart failure (CHF), new atrial fibrillation (Afib) or with rapid ventricular response (RVR), arrhythmias, stroke, and others. Peak cardiac biomarkers (troponin and brain natriuretic peptide [BNP]) were collected. Chart review was conducted by 2 physicians independently and consensus achieved.

Cardiac Risk Factors

**Figure:**
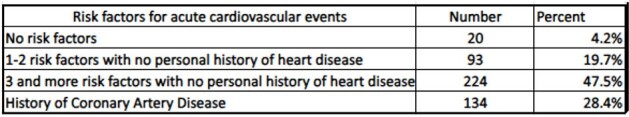

Cardiac Risk factors: HTN, hyperlipidemia, diabetes mellitus, family history of CAD in a first-degree relative, current smoking, BMI>30.

**Results:**

RSV was diagnosed in 471 hospitalized adults. The median age was 69 years, 59% were female and nearly all had underlying medical conditions (92%) with hypertension and diabetes mellitus most common (Figure 1). Cardiac risk factors are summarized in Table 1. During a high-risk period, 167 patients had 270 CVE (89% within 1 week) and 29 patients died. The most common complications were new or exacerbated CHF (27.5%) and Afib with RVR (12.9%), Figure 2. The rates of CVE during pre-RSV, high-risk, and late-risk periods were 0.34, 6.9, and 0.45 per person-years, respectively. Notably, the Incidence Rate Ratio (IRR) of CVE during the high-risk period was 19.9 (CI 15.5-25.9) compared to the pre-RSV period, p< 0.0001. IRR of CVE during late-risk period was 1.55 (CI 1.1-2.1) compared to pre-RSV period, p=0.003. Increased troponins occurred in 34% and high BNP in 62% of those tested.

**Figure:**
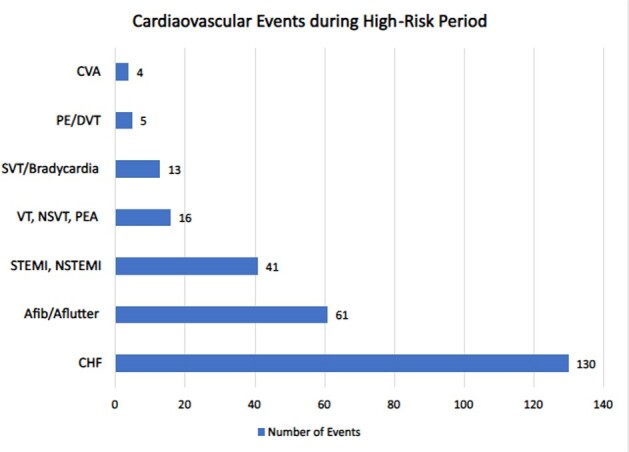

Total number of Cardiovascular Events (CE) was 270 events in 167 patients.

CHF – congestive heart failure new onset or decompensation requiring intervention; STEMI/NSTEMI – ST-Elevation Myocardial Infarction, Non-ST Elevation Myocardial Infarction; Afib/Aflutter – Atrial Fibrillation or Atrial Flutter new onset or with rapid ventricular response requiring intervention; SVT – supraventricular tachycardia requiring intervention; VT/NSVT/PEA – Ventricular Tachycardia, Non-sustained Ventricular Tachycardia, Pulseless Electrical Activity; PE/DVT – Pulmonary embolism, Deep Venous Thrombosis; CVA – Cerebrovascular Accident.

**Conclusion:**

RSV infection is associated with decompensation of chronic cardiac conditions as well as new CVEs with effects persisting past a traditional 28-day risk period. Prevented CVEs should be assessed in upcoming adult RSV vaccine effectiveness studies.

**Disclosures:**

**Edward E. Walsh, MD**, Enanta: Advisor/Consultant|Enanta: Honoraria|GSK: Advisor/Consultant|Janssen: Advisor/Consultant|Merck: Advisor/Consultant|Merck: Grant/Research Support|Pfizer: Advisor/Consultant|Pfizer: Grant/Research Support **Angela R. Branche, MD**, Cyanvac: Grant/Research Support|GSK: Advisor/Consultant|Merck: Grant/Research Support|Moderna: Grant/Research Support|Moderna: Honoraria|Novavax: Advisor/Consultant|Pfizer: Grant/Research Support|Sanofi: Honoraria|VaxCo: Grant/Research Support **Ann R. Falsey, MD**, ADMA: Board Member|BioFire Diagnostics: Grant/Research Support|CyanVac: Grant/Research Support|GSK: Advisor/Consultant|GSK: Travel support|Janssen: Grant/Research Support|Moderna: Advisor/Consultant|Moderna: Grant/Research Support|Pfizer: Advisor/Consultant|Pfizer: Grant/Research Support|Sanofi Pasteur: Advisor/Consultant|Sanofi Pasteur: Travel support|VaxCo: Grant/Research Support

